# Aggressive recurrent intestinal-type adenocarcinoma of the nasal cavity: a case report and literature review

**DOI:** 10.3389/fmed.2025.1510225

**Published:** 2025-04-15

**Authors:** YaoYao Chen, Jiawei Xu, Chengdong Yu, Haoyu Shi, Ling Luo, Dingbang Peng, Xiaofang Zhang, Wen Chen, Fei Le

**Affiliations:** ^1^Jiangxi Medical College, Nanchang University, Nanchang, China; ^2^Department of Pathology, Jiangxi Cancer Hospital, The Second Affiliated Hospital of Nanchang Medical College, Nanchang, China; ^3^Department of Breast Surgery, Jiangxi Cancer Hospital, The Second Affiliated Hospital of Nanchang Medical College, Nanchang, China; ^4^Department of Head and Neck Surgery, Jiangxi Cancer Hospital, The Second Affiliated Hospital of Nanchang Medical College, Jiangxi Clinical Research Center for Cancer, Nanchang, China

**Keywords:** intestinal-type adenocarcinoma, nasal cavity, immunotherapy, targeted therapy, recurrence, case report

## Abstract

Intestinal-type adenocarcinoma (ITAC) of the nasal cavity is a rare, aggressive tumor that presents unique therapeutic challenges, particularly in cases of recurrent disease with intracranial extension. We present the case of a 54-year-old male with recurrent ITAC who demonstrated a remarkable response to a novel triple-therapy approach. This patient lacked the typical risk factors, such as occupational exposure to wood or leather dust, making this case particularly unusual. After failing conventional treatments and experiencing multiple recurrences with progressive intracranial invasion, the patient received an innovative combination of immunotherapy (Toripalimab), targeted therapy (Anlotinib), and chemotherapy (S-1). This unprecedented therapeutic combination resulted in significant tumor volume reduction (from 53.9 × 42.7 mm to 38.9 × 18.8 mm) and marked improvement in neurological symptoms, particularly diplopia. To our knowledge, this represents the first case report of successful implementation of this specific triple therapy regimen for recurrent ITAC with intracranial extension.

## Introduction

Intestinal-type adenocarcinoma (ITAC) of the nasal cavity is an exceedingly rare malignant tumor, mainly occurring in the nasal cavity and paranasal sinuses. This type of adenocarcinoma is often associated with occupational exposure to wood and leather dust (1), as well as specific genetic mutations such as TP53 (2). Histopathologically, ITAC resembles colorectal adenocarcinomas but exhibits unique molecular characteristics that complicate its diagnosis and treatment (3). ITAC presents unique therapeutic challenges, particularly in cases of recurrence with intracranial extension.

While conventional treatments, including surgery and radiotherapy, remain the standard of care, management of recurrent disease lacks standardized protocols. Given the rarity and complexity of ITAC, particularly in cases with intracranial extension, finding new treatments is critical. Here, we present a case involving a novel therapeutic combination of immune checkpoint inhibition, targeted therapy, and chemotherapy to treat recurrent ITAC. This report includes detailed histological, molecular, and radiological evidence supporting this treatment strategy, alongside a comprehensive review of current therapeutic approaches. This case emphasizes the importance of a multidisciplinary approach to managing this rare and persistent malignancy (4, 5).

## Case report

A 54-year-old male with no significant medical history presented in October 2016 with intermittent epistaxis and right nasal obstruction. The patient had no known occupational exposure to wood or leather dust, nor a history of smoking or alcohol use.

In October 2017, a biopsy at Jiangxi Provincial People’s Hospital revealed adenocarcinoma. Further evaluation at the Otolaryngology Hospital affiliated with Fudan University confirmed the diagnosis of ITAC in the right nasal cavity (pT2N0M0, Stage II). Gastroenteroscopy ruled out a primary gastrointestinal source, and Epstein–Barr virus testing excluded nasopharyngeal carcinoma. A PET-CT scan at Shanghai Zhongshan Hospital identified a localized right nasal adenocarcinoma with no evidence of systemic metastasis.

The patient underwent endoscopic resection of the right nasal tumor in October 2017. Postoperative pathology confirmed ITAC, achieving negative margins (R0 resection), followed by adjuvant radiotherapy (total dose: 60 Gy in 30 fractions).

**Figure 1:**
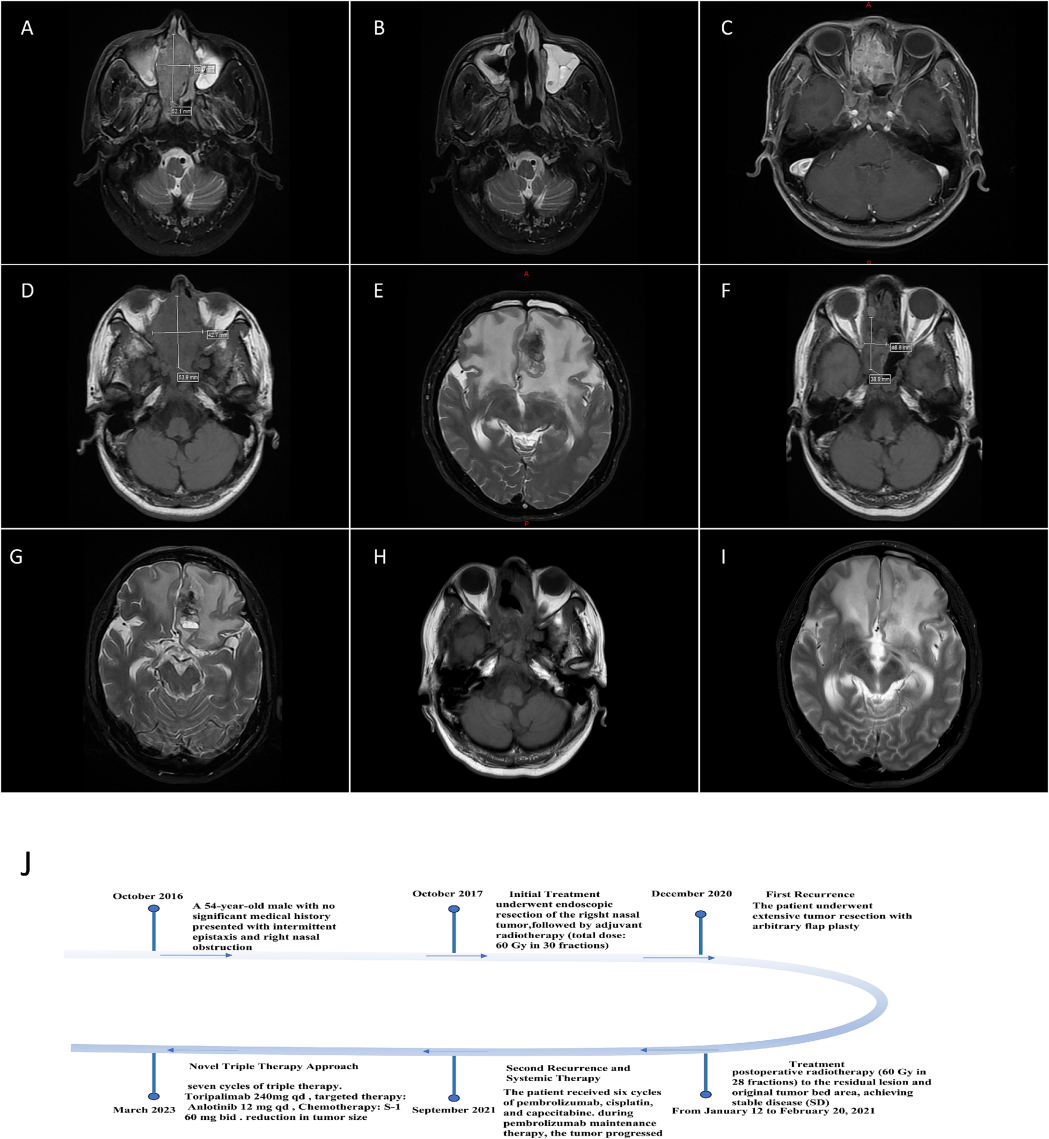
MRI Imaging of ITAC. **(A)** Preoperative MRI: this image demonstrates a moderately enhancing mass within the right nasal cavity with poorly defined margins. The lesion extends posteriorly to involve the nasopharyngeal cavity, causing narrowing. There is a leftward deviation of the nasal septum, and the signal intensity within the lesion is heterogeneous. **(B)** Postoperative MRI: the image shows the absence of the right middle and superior nasal conchae, as well as a partial resection of the nasal septum. Most of the irregular soft tissue mass previously occupying the right nasal cavity has been surgically removed, with residual post-surgical changes visible. **(C)** MRI after surgery and radiotherapy: the lesions in the ethmoidal sinus at the upper margin of the operative area had increased in size compared to before, with invasion of the frontal lobe base and the anterior part of the brain. Tumor recurrence with a high likelihood of invasion into adjacent structures was considered. **(D)** The mass in the nasal cavity surgical area has increased in size compared to the previous examination, with an expanded range of intracranial invasion. The extent of invasion has also increased in the medial walls of both orbits, the orbital apices, and both optic nerves. **(E)** The extent of perilesional brain edema has increased. **(F)** Follow-up after comprehensive treatment for recurrent nasopharyngeal malignancy: The previous findings of middle turbinate defect and partial absence of the nasal septum remain unchanged. The soft tissue mass in the surgical area has decreased in size, with an ill-defined border. The invasion of the frontal lobe and basal meninges at the upper margin of the lesion has shown some improvement. There is also improvement in the invasion of the clivus, medial walls of both orbits, orbital apices, optic nerves, pterygoid processes, and the internal and external pterygoid muscles on both sides. **(G)** Reexamination MRI: the scope of cerebral edema was reduced. **(H)** MRI review in August 2023 indicated a slight enlargement of the soft tissue mass in the operative area. **(I)** The degree of bilateral cerebral edema has worsened compared to the previous imaging examination. **(J)** Patient Treatment Schedule.

**Figure 2:**
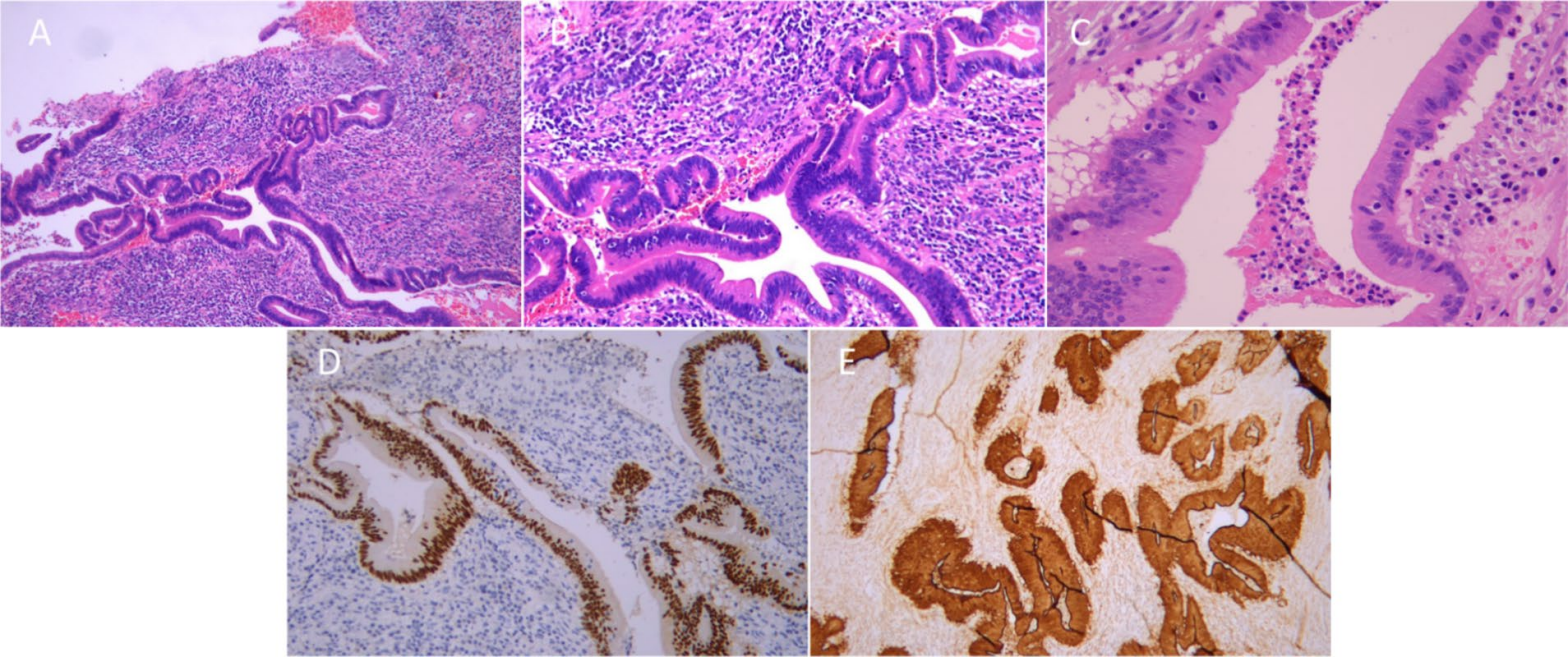
Comprehensive Pathological Analysis of Recurrent ITAC: H&E Staining and Immunohistochemical Findings. **(A–C)** The proliferation of tumor cells was glandular tubular, the nucleus was rod-shaped, the cell layers were increased, the cells were piled up and squeezed, the cell cytoplasm was rich and red stained, and the cell apoptosis and nuclear division images could be seen. Fibrous connective tissue hyperplasia and a large number of lymphocyte aggregation were observed in tumor interstitium. Immunohistochemical stained sections (original magnification ×200). CDX2 **(D)** and Villin **(E)** are positive.

The patient remained disease-free until December 2020, when nasopharyngoscopy revealed complete obstruction of the right nasal cavity with friable, pale tissue prone to bleeding. MRI demonstrated an enhancing mass (52.1 × 28.6 mm) in the right nasal cavity extending to the nasopharynx (Figure 1A). Comprehensive systemic imaging ruled out other primary tumors.The patient underwent extensive tumor resection with arbitrary flap plasty. Postoperative MRI confirmed partial removal of the mass (Figure 1B). Histopathological examination revealed invasive glandular tubular patterns with marked nuclear pleomorphism, frequent mitoses, and a high Ki-67 proliferative index (30%), indicating strong proliferative activity. No evidence of lymphovascular invasion or perineural invasion was observed (Figures 2A–C). Immunohistochemical (IHC) analysis showed CK20 positivity, CK7 negativity, strong CDX2 expression, and microsatellite stability (MSS) (Figures 2D,E). PD-L1 expression was negative, and p53 analysis revealed wild-type expression. HER2 was weakly positive in 40% of tumor cells. Postoperative pathology confirmed ITAC. The tumor was staged as rT3N0M0, Stage II. From January 12 to February 20, 2021, the patient received postoperative radiotherapy (60 Gy in 28 fractions) to the residual lesion and original tumor bed area, achieving stable disease (SD).

In September 2021, MRI revealed tumor recurrence with invasion into the frontal lobe and anterior brain, accompanied by cerebral edema (Figure 1C). The patient received six cycles of pembrolizumab, cisplatin, and capecitabine, achieving stable disease. However, during pembrolizumab maintenance therapy, the tumor progressed.

In March 2023, the patient presented with diplopia and fatigue. MRI revealed significant tumor enlargement (53.9 × 42.7 mm), accompanied by extensive intracranial invasion (Figures 1D,E). The progress of the patient was considered according to the results of the examination and general discussion. The diagnosis confirmed recurrent malignant tumor of the right nasal cavity (stage T4N0M0 IV). Following multidisciplinary team (MDT) consultation, a novel triple therapy regimen was initiated: Immunotherapy: Toripalimab 240 mg qd (D1-14, Q3W), targeted therapy: Anlotinib 12 mg qd (D1-14, Q3W), Chemotherapy: S-1 60 mg bid (D1-14, Q3W).

During treatment, the patient developed fever and hiccups, which may be related to the treatment regimen. After symptomatic treatment such as physical cooling, antiemesis, antihiccup and sodium supplementation, the symptoms were effectively controlled and the tolerance of treatment was ensured.

After seven cycles of triple therapy, MRI demonstrated a marked reduction in tumor size (38.9 × 18.8 mm) and improvement in cerebral edema (Figures 1F,G). The patient reported significant symptom relief, including resolution of diplopia. For post-treatment management, patients should undergo weekly blood routine examinations and biochemical assays to assess hematological and biochemical parameters, and timely symptomatic treatment should be provided for abnormalities such as cell reduction or electrolyte imbalance. Regular imaging tests are recommended to assess tumor response and detect recurrence. Follow-up imaging in August 2023 showed slight tumor enlargement accompanied by increased bilateral frontal lobe edema (Figures 1H,I), but the patient remained clinically stable. The triple therapy regimen was well-tolerated, with no grade 3–4 adverse events reported. Furthermore, we developed a comprehensive patient treatment timeline that systematically delineates the therapeutic interventions and clinical progression (Figure 1J).

## Discussion

The presented case of ITAC of the nasal cavity emphasizes the complexity and aggressive nature of this rare malignancy. In the context of relevant literature, the treatment methods and recurrence of nasopharyngeal enteric adenocarcinoma were summarized (Table 1). The purpose is to offer clinicians with important findings into the diagnosis and treatment of this rare disease.

**Table 1:** Case reports on ITAC of the nasal cavity.

Author	Year	Age	Gender	Diagnosis	Organism	Treatment	Recurrence
Belli et al. (17)	2014	31	Male	High-grade intestinal-type adenocarcinoma	Nasal cavity	Endoscopic excision	No recurrence after 3 years
Izzo et al. (19)	2016	84	Male	Sinonasal adenocarcinoma	Nasal cavity	Chemoradiotherapy	Not specified
Moor et al. (20)	2004	76	Female	Mucinous intestinal-type adenocarcinoma	Sinonasal tract	Transfacial nasoethmoidectomy, reconstruction	No recurrence after 20 months
Clausa et al. (21)	2019	68	Male	Sinonasal intestinal-type adenocarcinoma	Sinonasal tract with intracranial invasion	Cisplatin-5-fluorouracyl	Not specified

Nasal cavity and paranasal sinus carcinomas constitute approximately 0.2 to 0.5% of all malignant tumors in the human body, with the majority being squamous cell carcinomas (6). Intestinal-type sinonasal adenocarcinoma is a rare subtype of epithelial tumors of the nasal cavity and paranasal sinuses, accounting for only 8 to 25% of all malignant sinonasal tumors (7). This type of tumor typically originates from the inferior or middle nasal concha and is often associated with prolonged occupational exposure to wood dust (8). According to the International Agency for Research on Cancer, prior and ongoing exposure to certain toxic chemical and physical substances plays a central role in the pathogenesis of this type of cancer. This case involves a rare occurrence of nasal cavity adenocarcinoma in a patient who had no prior exposure to known risk factors.

ITAC’s develop especially in men, demonstrated by a male: female ratio of 21:1, and the mean onset is between 60 and 65 years of age (9). This tumor exhibits histological characteristics similar to colorectal adenocarcinoma. But has distinct molecular features, including overexpression of certain biomarkers like CK20 and CDX2, which can aid in diagnosis (10). ITAC is the primary subtype of nasal cavity adenocarcinoma. Its most common symptoms include epistaxis, unilateral nasal obstruction, and rhinorrhea, making it difficult to distinguish from chronic sinusitis. ITAC is especially difficult due to its nonspecific symptoms, which often lead to delayed diagnosis and mismanagement.

ITAC is characterized by extensive tumor invasion into adjacent structures, including the eyes, optic nerves, optic chiasm, frontal and temporal lobes, and brainstem, while lymphatic or hematogenous metastasis is relatively rare. The standard treatment consists of complete surgical resection combined with postoperative adjuvant radiotherapy (11). Although the use of endoscopic endonasal surgery has somewhat improved outcomes, the overall prognosis remains poor, with a 5-year overall survival rate of 68% and a local recurrence rate of 30% (12–14). Among recurrences, 88% are local, and about 9% are distant metastases (9). In this case, a 54-year-old male patient was initially diagnosed with ITAC and underwent surgery and radiotherapy but experienced multiple recurrences over several years, stressing the persistent nature of ITAC and the challenges in achieving long-term remission. Adjuvant radiotherapy can reduce the risk of local recurrence, while the integration of systemic therapies, such as chemotherapy, targeted therapy, or immunotherapy, may further improve local control, decrease distant metastases, and enhance survival in cases of unresectable disease.

To further refine therapeutic strategies, molecular and immunohistochemical (IHC) analyses provide critical findings into the tumor’s biological characteristics and potential therapeutic targets. In this case, IHC revealed strong staining for villin, CK20 positivity, CK7 negativity, and strong CDX2 expression, confirming the diagnosis of ITAC. A Ki-67 proliferative index of 30% indicated high tumor proliferation. Microsatellite instability (MSI) testing confirmed microsatellite stability (MSS), while p53 analysis showed partial positivity with wild-type expression, suggesting no significant p53 mutations. PD-L1 expression was negative, complicating the use of immune checkpoint inhibitors.

It is unclear how the specific choice of immunotherapy is related to histological histotype and whether there is a molecular profile that underlies the therapeutic choice. In our case, despite the absence of predictive biomarkers for immunotherapy efficacy, such as PD-L1 positivity or mismatch repair (MMR) deficiency, the tumor’s aggressiveness and limited systemic options justified exploring immunotherapy. Studies suggest that combining PD-1 inhibitors with radiotherapy may improve outcomes in MSS colorectal cancers, providing a rationale for their application in MSS ITAC (15). The successful response observed in our patient, despite negative PD-L1 expression and microsatellite stability, suggests that traditional biomarkers alone may not fully predict immunotherapy efficacy in ITAC. This stressing the need for further research to identify more precise molecular profiles that could better predict treatment response in these rare tumors. Weak HER2 positivity was observed in 40% of tumor cells, but it is not currently considered a viable therapeutic target. Additionally, while MUC2, a marker of intestinal differentiation, was not assessed, its evaluation could aid future diagnostics. Recent research has identified tumor budding (TB) as a critical prognostic factor in ITAC. High TB (>4 buds) is associated with poor outcomes, including increased recurrence and reduced survival. Incorporating TB evaluation into clinical practice could help identify high-risk patients who may benefit from more aggressive or innovative therapeutic approaches (16). Notably, a novel triple therapy regimen combining Toripalimab, Anlotinib, and S-1 demonstrated promising efficacy in controlling both local disease and intracranial extension, suggesting potential synergistic effects among these agents and offering a new avenue for treatment in advanced ITAC. To advance the understanding and clinical application of triple therapy (immunotherapy, targeted therapy, and chemotherapy) in sinonasal malignancies, future efforts should prioritize conducting prospective clinical trials to establish its efficacy and safety.

The recurrence of ITAC in the nasal cavity observed in this case emphasizes the malignancy’s aggressive nature and its propensity for high recurrence, as well documented in the literature. Despite the initial aggressive treatment, including surgical resection and radiotherapy, the recurrence in our patient stressing the limitations of these conventional approaches for achieving long-term disease control (17). This aligns with the findings of Hoeben et al., who emphasize the importance of exploring molecular-targeted therapies to enhance treatment efficacy (12). Recent research has focused on molecular pathways such as EGFR, MET, and H-RAS, which may offer targets for new therapies (18).

## Conclusion

This case presents a promising therapeutic strategy to manage aggressive recurrent ITAC with intracranial extension. Triple therapy, which combines immunotherapy, targeted therapy, and chemotherapy, successfully offers a fresh treatment option for patients who have few alternatives. Our team plans to further explore this approach through prospective clinical trials to confirm its effectiveness in a broader patient group.

## Data Availability

The original contributions presented in the study are included in the article/supplementary material, further inquiries can be directed to the corresponding authors.
